# Chinese Medicinal Herbs in the Treatment of Diabetic Cognitive Impairment: A Systematic Review and Meta-Analysis

**DOI:** 10.1155/2018/7541406

**Published:** 2018-09-13

**Authors:** Bin Yan, Jingbo Wang, Zhigang Xue, Guoqing Tian

**Affiliations:** ^1^Department of Traditional Chinese Medicine, Peking Union Medical College Hospital, Chinese Academy of Medical Sciences, China; ^2^Department of General Surgery, Peking Union Medical College Hospital, Chinese Academy of Medical Sciences, China

## Abstract

**Background:**

Diabetic cognitive impairment (DCI), a serious complication of diabetes mellitus (DM), is gaining more acceptance and attention. The learning and memory function of diabetics always decreases. Traditional Chinese medicine (TCM) has been demonstrated to be effective in treating the symptoms in China, and thereinto Chinese medicinal herbs (CMH) are the most widely used. The objective of the present study was to review and analyze the existing data about reducing the symptoms in CMH treatment for DCI.

**Methods:**

Electronic literature databases (PubMed, EMBASE, CNKI, SinoMed, and Wan fang) were searched for randomized controlled trials conducted in China, comparing CMH with western medicines in the treatment of DCI, up to April 1, 2018. We applied standard meta-analytic techniques to analyze data from papers that reached acceptable criteria.

**Result:**

Nine randomized controlled trials (n = 576) on CMH were included. We found moderate evidence that CMH used alone or in combination with western medicines was more effective than western medicines alone in reliving the symptoms for DCI (total effective rate, odds radio (OR) = 4.64 (2.60, 8.29), and 95% confidence interval, P<0.00001). Besides, CMH along or in combination with western medicines showed more beneficial effects on Montreal Cognitive Assessment (MoCA) scale (mean difference (MD) = 1.31(0.75, 1.87), P<0.00001), Mini-Mental State Examination (MMSE) scale (MD = 2.07 (0.86, 3.28), P<0.00001, and TCM symptom score (TCMSS) (MD = -4.89 (-8.44, -1.34), P = 0.007). Most of the included studies showed that there was not a significant difference in the adverse events.

**Conclusions:**

These findings demonstrated that CMH used alone or in combination with western medicines were apparently better than western medicines alone in the treatment of DCI. Because of the poor quality of the studies that were available for the present meta-analysis, further researches are still needed to support these early findings.

## 1. Introduction

With the improvement of living standards, the acceleration of aging process, and the changes in lifestyle, the patients with diabetes mellitus (DM) are increasing rapidly in China [1]. An epidemiological survey showed that the prevalence of diabetes and prediabetes in adults was 9.7% and 15.5% in 2010 [2], and the 8^th^ edition IDF Diabetes Atlas, released by the International Diabetes Federation, displayed a fact that the number of diabetics was about 114 million by 2017[3]. DM can lead to a variety of chronic complications [4] that are a serious threat to human health. In recent years, the cognitive impairment associated with DM has been paid more and more attention, and it mainly refers to the decline of cognitive function in diabetes patients [5–9], which is characterized by slow thinking, the decline of learning and memory, dependence enhancement, etc.

Diabetic cognitive impairment (DCI) is mainly caused by the damage of DM to the central nervous system, and modern medicine thinks that it may be related to hyperglycemia [10, 11] insulin resistance [12, 13], oxidative stress [14], neuroinflammation [15, 16], etc. However, there is no specific treatment yet. To find some effective therapeutic methods, many researchers have turned their attention to complementary and alternative medicine, such as Traditional Chinese Medicine (TCM).

There is no definite disease name of DCI in TCM literature, but according to its clinical characteristics and performance, it is equivalent to “Xiaoke” combined with “forgetfulness”. Compared with modern medicine, TCM focuses more on the improvement of subjective feelings of patients and adheres to the individual treatment of differentiation of symptoms and signs [17], so that the patients are more likely to accept the treatment and establish the confidence of rehabilitation. However, just because of syndrome differentiation and treatment, TCM lacks unified treatment plan and clinical curative effect standard [18], which hinders the spread of TCM worldwide. Consequently, the research on the treatment of DCI with TCM is mainly carried out in China, still lacking worldwide clinical data.

Chinese medicinal herb (CMH) is the typical representatives of TCM, and it has been used to treat DCI in China. Various studies including both in vitro and in vivo experiments have verified the efficacy of CMH regarding the management of DCI. The evidences from the animal researches have suggested that CMH could reduce the apoptosis of hippocampal neurons, which are responsible for memory storage, possibly by upregulation of Bcl-2 and downregulation of Bax [19], and repair the synaptic structure, and increase the expression of insulin-like growth factor I [20]. However, there is no a systematic review about CMH in the treatment of DCI as so far, and non-TCM experts may face problems to perform further research in the related field. In this report, we employed a meta-analysis to determine “whether CMH is more beneficial in DCI as compared with western medicines alone”.

## 2. Methods

### 2.1. Data Source and Search Strategy

Studies were identified by a comprehensive search in the following 5 databases: PubMed, EMBASE, China National Knowledge Infrastructure (CNKI), Sinomed, and Wan Fang database. The search was conducted between the inception of each database and April 1, 2018. Searches were conducted by combining the following terms: (“diabetic cognitive impairment” OR “diabetic cognitive dysfunction” OR “diabetic encephalopathy” OR (“Diabetes Mellitus” and “cognitive dysfunction”)) AND (“traditional Chinese medicine” OR “Chinese medicine herbs” OR “herbal medicine”) AND (“random”). While searching Chinese databases, we adopted the similar search strategy with Chinese terms.

### 2.2. Inclusion Criteria

#### 2.2.1. Type of Study

The type of study was randomized controlled trial (RCT) published in the English or Chinese language regardless of whether there is single blind, double blind, or nonblind.

#### 2.2.2. Study Objects

Clearly diagnosed patients of DCI: (1) the subjects included are in line with the 1999 World Health Organization (WHO) diagnostic criteria for DM or the 1997 American Diabetes Association (ADA) diagnostic criteria; (2) the subjects included are in line with diagnostic criteria for cognitive impairment: a reference to the Hasegawa Dementia Scale or Mini-Mental State Examination (MMSE).

#### 2.2.3. Interventions

Usually conventional treatment is combined with the following treatments: (1) the test group was treated with CMH, and the control group was treated with western medicine alone; (2) the test group was treated with CMH combined with western medicine, and the control group was treated with western medicine alone; (3) the types of western medicine were western conventional medicines used in the treatment of DCI, such as adopting nimodipine, huperzine A; (4) both groups were given conventional treatment to control blood glucose.

#### 2.2.4. Outcome Measures

At least one of the following outcomes was evaluated: (1) total effective rate: if more than 30% of the symptoms and signs were reduced, the therapy was effective or was ineffective; (2) Montreal Cognitive Assessment (MoCA): the higher score represents the better cognitive function; (3) MMSE: tsame as MoCA, the higher score represents the better cognitive function; (4) TCM symptom score (TCMSS): contrary to MoCA, the lower score represents the less symptoms.

### 2.3. Exclusion Criteria

We excluded the following trials: (1) other methods of treatment as intervention measures; (2) nonrandom or uncontrolled trials; (3) cognitive impairment caused by trauma, cerebral hemorrhage, cerebral infarction, chronic systemic disease, or mental disease; (4) case reports, expert experiences; (5) nonclinical researches like animal experiments etc.

### 2.4. Data Extraction

A standardized data sheet was utilized to extract the data in a uniform way from each of the eligible studies, including details about the authors, the year of publication, study design, and total sample size. Primary outcomes included assessment of efficacy and the change of cognitive function score (e.g., MoCA, MMSE, and TCMSS).

### 2.5. Assessment of Study Quality

We assessed the quality of included studies using the five-point Jadad scale [21]. Three components associated with the risk of bias were assessed: sequence generation, blinding, and reason for dropping out. Specific scoring criteria were as follows: when the study offered a detailed description of randomization, such as adopting random number table, two points were given; if the RCT offered only general comments without an exact description, one point was given; no random allocation, no scoring. When the study used a completely consistent placebo or similar method, two points were given; when the test referred to blind method but did not describe it, one point was given; no use of blinding without scoring. When the specified number and reasons for drop-outs by each subject group were provided, one point was given, or it would be zero; and a definite statement was needed when there were no drop-outs. If the total score was ≥3 points, the paper was considered of high quality. We also assessed allocation concealment as adequate, unclear, or no [22].

### 2.6. Statistical Methods

Statistical analyses were conducted using Review Manager 5.3, which was provided freely by the Cochrane cooperation net for meta-analysis [23]. The data collected were divided into dichotomous and continuous variables and Odds Ratio (OR) and Mean Difference (MD) were used as effect values, respectively. The confidence interval was set as *α* = 0.05 for all analyses. Variability among studies in a systematic review would be termed heterogeneity, and, according to the heterogeneity level, individual effect sizes were composed to generate an overall effect size using a fixed or random effects model. Heterogeneity was assessed using Cochrane's P and I^2^, which calculated the proportion of variation attributed to heterogeneity. P > 0.10 indicated that the studies had homogeneity, so fixed effects model was used; otherwise the random effect model was used. Besides, if I^2^ ≤ 50%, the homogeneity of the studies was acceptable, when I^2^ > 70%, potential source of heterogeneity was identified by sensitivity analyses.

## 3. Result

### 3.1. Study Selection and Study Characteristics

**Figure 1:**
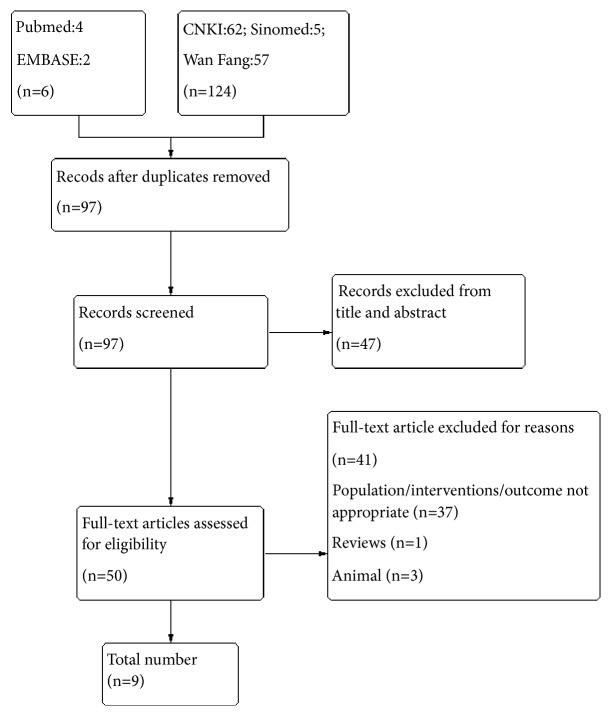
Selection strategy.

After searching the database systematically, a total of 130 articles were identified, and the title and abstracts were screened. 50 full text articles were reviewed, and 9 [24–32] papers met the inclusion criteria (Figure 1). The 9 included RCT studies involved 576 subjects, and the information of the 9 studies was presented in Tables 1 and 2. All of these 9 studies were in Chinese, and 7 Chinese medicine formulas were used in the included studies. The 5 test groups [25, 27, 28, 30, 31] used CMH alone, and the rest 4 test groups [24, 26, 29, 32] were in combination with western medicines.

All the researches were referred to using randomization, but only 3 studies, Cai 2016 [24], Jin 2013 [26], Zhao 2014 [32], adequately used random number table method for randomization, and the rest of the researches, though used randomization for grouping, were not referred to any specific methods. All of the included researches did not mention allocation concealment. 4 studies [25, 26, 28, 32] alluded to blinding, but did not refer to the particular methods. 3 studies [25, 29, 32] mentioned the follow-up description. There was no significant difference in age, gender, education levels, and disease duration in all the researches and the studies are comparable. Among the studies, 5 researches [25–28, 31] described the total effective rate, 4 researches [26, 27, 29] described the MoCA score, 7 researches [24, 25, 27–31] described the MMSE score, and 5 researches [24, 26, 27, 30, 32] described the TCMSS score.

### 3.2. Quality of the Included Studies

Most of the researches scored no more than 2 points (Table 3), and only 3 studies [25, 26, 32] scored more than 2 points, which meat that the quality of the majorities was low. As shown in the table, given the characteristics of CMH forms and taste, it was difficult to carry out allocation concealment and blinding.

### 3.3. CMH in DCI

#### 3.3.1. CMH versus Western Medicine on Total Effective Rate

**Figure 2:**
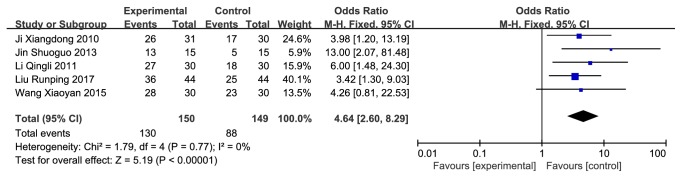
Forest plot depicting the total effective rate.

**Figure 3:**
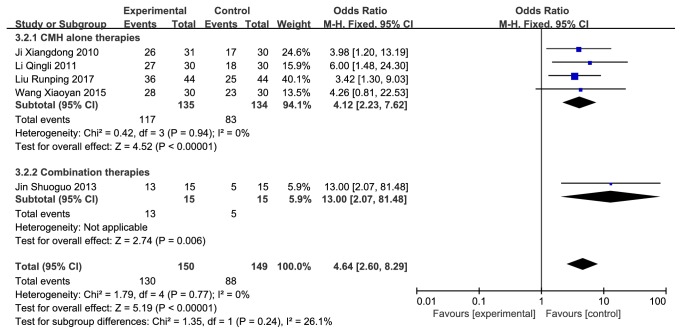
Forest plot depicting the total effective rate subgroup.

There were 5 studies (n = 279 subjects) being identified [25–28, 31]. We observed significant differences on total effective rate in favor of CMH used alone or in combination with western medicines compared with the western conventional medicines alone (odds radio (OR) = 4.64 (2.60, 8.29), 95% confidence interval, P<0.00001, I^2^ = 0%) (Figure 2). Besides, 4 of the 5 studies were using CMH alone therapies, so we did a subgroup analysis according to whether it was a combination therapy (Figure 3).

#### 3.3.2. CMH versus Western Medicine on MoCA

**Figure 4:**
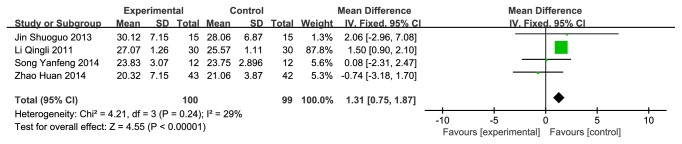
Forest plot depicting the MoCA.

**Figure 5:**
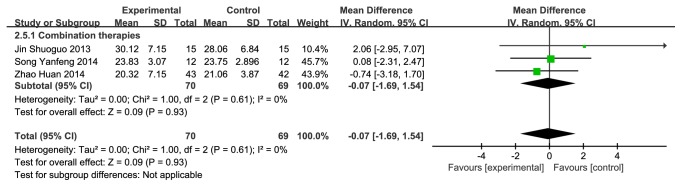
Forest plot depicting the MoCA subgroup.

There were 4 studies (n = 199 subjects) identified [26, 27, 29, 32]. Compared with western conventional medicines, CMH used alone or combination therapies displayed significant differences on MoCA (mean difference (MD) = 1.31(0.75, 1.87), P<0.00001, I^2^ = 29%) (Figure 4), which indicated that CMH used alone or combination therapies had an advantage in improving the MoCA score. A subgroup, including 3 studies [26, 29, 32], was made to compare the combination therapies with the CMH alone therapies on MoCA (MD = -0.07 (-1.69, 1.54), P = 0.93, I^2^ = 0%) (Figure 5), which showed no significant difference.

#### 3.3.3. CMH versus Western Medicine on MMSE

**Figure 6:**
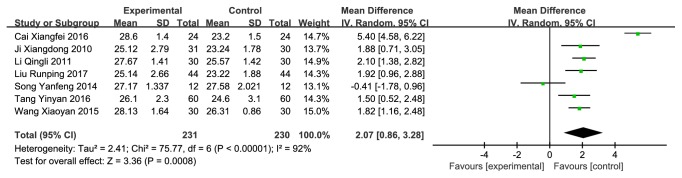
Forest plot depicting the MMSE.

**Figure 7:**
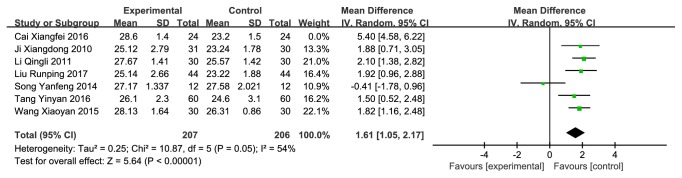
Forest plot depicting the MMSE after sensitivity analysis.

**Figure 8:**
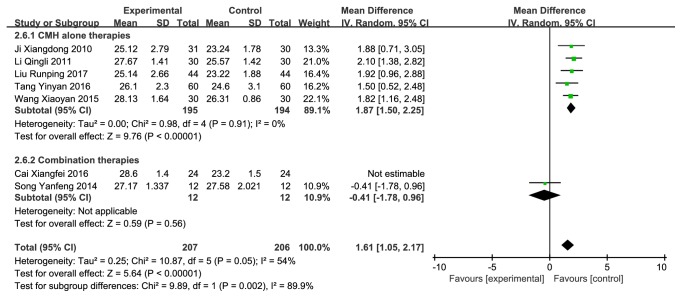
Forest plot depicting the MMSE subgroup.

7 studies [24, 25, 27–31] (n = 461 subjects) compared CMH used alone or combination therapies with western conventional medicines on improving MMSE scale, of which the integrated results showed that CMH used alone or combination therapies was clinically superior to western medicines alone on MMSE score improvement (MD = 2.07 (0.86, 3.28), P<0.00001), but there were high heterogeneities across studies (I^2^ = 92%) (Figure 6). A sensitivity analysis made difference and declined the heterogeneities (I^2^ = 62%) (Figure 7) after removing a study whose data was obviously different with the others. Then we also did a subgroup analysis in the light of whether it was a combination therapy (Figure 8), and the result showed significant difference.

#### 3.3.4. CMH versus Western Medicine on TCMSS

**Figure 9:**
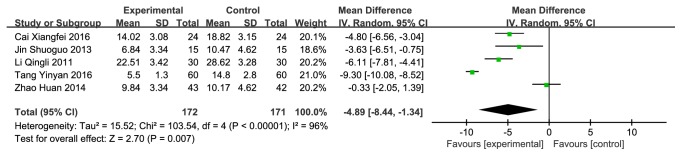
Forest plot depicting the TCMSS.

**Figure 10:**
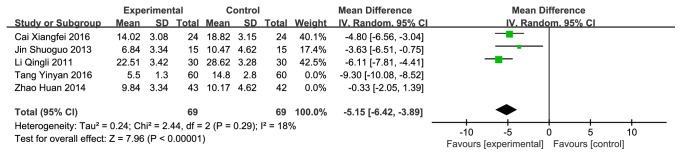
Forest plot depicting the TCMSS after sensitivity analysis.

**Figure 11:**
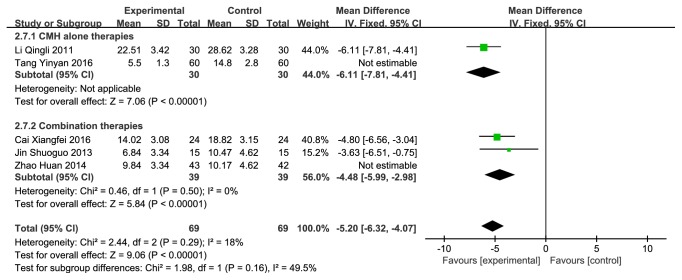
Forest plot depicting the TCMSS subgroup.

5 trails [24, 26, 27, 30, 32] (n = 343 subjects) compared CMH used alone or combination therapies with western conventional medicines alone, and we observed significant differences on improving TCMSS (MD = -4.89 (-8.44, -1.34), P = 0.007, I^2^ = 96%) (Figure 9). In sensitivity analyses limited the 3 trails [24, 26, 27], the MD was -5.15 (95%CI, -6.42 to -3.89, I^2^ = 18%) (Figure 10). Also a subgroup analysis was needed whether it was a combination therapy (Figure 11), and the result showed significant difference.

#### 3.3.5. Publication Bias

Because of the scarcity of researches, we cannot judge the publication bias.

## 4. Discussion

TCM has a variety of ways in treating disease. After searching, it was found that CMH is the main form of treatment in curing DCI. This finding could also indicate that CMH was still the key therapy as adopting TCM. So the included researches focused on CMH in the study. We failed to find a study about CMH in the treatment of DCI in PubMed or EMBASE, which revealed that CMH were mainly adopted in China but were not well understood in western countries.

There is no uniform standard for the evaluation of cognitive function at present. These 9 studies related to several varieties of evaluation methods, such as MoCA, MMSE, daily living scale (ADL), Hasegawa Dementia Scale (HDS), and TCMSS, and we chose several of them, which were used more than 2 times, as indicators. MoCA, MMSE, and TCMSS all reflected the cognitive function to a certain extent. All the treatments in the studies showed positive effectiveness compared with baseline measurements. Compared with western conventional medicines, CMH used alone or in combination with western medicines might be more effective in improving DCI, which showed higher efficiency and better MoCA, MMSE, and TCMSS scores. Since 4 of the 9 trial groups were in combination therapies, we did subgroup analysis when analyzing every indicator. From Figures 3, 5, 8, and 11, we found that both combination therapies and TCM used alone were superior to western conventional medicines on the total effective rate and the TCMSS. As for MoCA and MMSE, there was no significant difference between combination therapies and western conventional medicines, but the CMH used alone still showed its advantages. We thought the main reason for this was that there were different combination and CMH therapies in different studies, and there were also not sufficient research samples. So we could not draw a conclusion summarily that CMH used alone was superior to combination therapies. We hope to analyze the advantages and disadvantages again between them in the future when there are enough rigorous researches. In general, we can see a trend that combination therapies and CMH used alone therapies are better than western conventional medicines. However, when it comes to CMH, we found that different individual studies might adopt different formulation. This is because of the unique characteristics of traditional Chinese Medicine: syndrome differentiation and treatment. But from these prescriptions we can also found something in common. TCM theories believe that kidney stores the human essence and affects the production of marrow [33]. When the marrow is full, the brain functions well. Thus almost all prescriptions contained tonifying kidney medicine. Additionally, high heterogeneities were found when we evaluated the effect of treatments on MMSE and TCMSS, and it fell to an extent that we can accept after eliminating one or two studies. This result may be due to the small sample sizes and low strength of the evidence in the studies.

A standard clinical trial should observe and refer to whether there were adverse effects or not. Though 4 of the included studies did not clearly mentioned about the adverse effects, the other 5 studies have showed that there were no significant difference on adverse effects between the CMH used alone or in combination with western medicines and western conventional medicines. We could draw the conclusion that CMH is safe for the treatment of DCI.

With regard to clinical importance, we found that most of the study sizes were small. With respect to the strength of evidence, we found that most of the comparisons had low strengths of evidence. We believe that the causes for this variation are mainly derived from the following reasons: (1) the quality of the individual studies was lowered because most of the trials failed to include a blinded control group, which was due to the characteristics of the interventions themselves; (2) the sample sizes of some trials were small, which would lower the strength of the evidence directly by one level; (3) inconsistency (i.e., different directions of effects or high heterogeneity (I^2^ ≥ 50%)) exists between trials. Obviously, inconsistency of study results in a meta-analysis and reduces the confidence of recommendations regarding treatment.

## 5. Conclusion

Although there were many deficiencies in our review, the major strength of our study was the coverage of all aspects of CMH in individuals with DCI. In conclusion, this analysis indicates that CMH used alone or in combination with western medicines plays a significant role in improving DCI, which shows a higher total effective rate and better MoCA, MMSE, and TCMSS scores.

Nevertheless, considering the sparse studies and inferior methodological quality of the individual trials, we propose that sham therapies need to be designed as soon as possible to improve the strength of evidence. More large samples, high quality, and multicenter researches are needed to provide more scientific and accurate decision-making basis and reference for the clinical work.

## Figures and Tables

**Table 1:** Basic information of 10 RCT studies.

No.	Authors	Year	Sample		Prevention	
			TG	CG	TG	CG
1 [24]	CAI Xiangfei	2016	24	24	Yishen Huoxue Fang +Routine western medicine	Routine western medicine
2 [25]	JI Xiangdong	2010	31	30	Bushen Quyu Yizhi Decoction	Nimodipine
3 [26]	JIN Shuoguo et al.	2013	15	15	Bushen Huoxue Kaiqiao Fang + Nimodipine	Nimodipine
4 [27]	LI Qingli et al.	2011	30	30	Zhinao Granule	Huperzine A
5 [28]	LIU Runping	2017	44	44	Bushen Quyu Yizhi Decoction	Nimodipine
6 [29]	SONG Yanfeng et al.	2014	12	12	Jin Maitong + Routine western medicine	Routine western medicine
7 [30]	TANG Yinyan et al.	2016	60	60	Tang Naoqing Granule	Nimodipine
8 [31]	WANG Xiaoyan	2015	30	30	Bushen Jiannao Fang	Nimodipine
9 [32]	ZHAO Huan et al.	2014	43	42	Bushen Huoxue Kaiqiao Fang +Aspirin	Aspirin

TG, test group; CG, control group.

**Table 2:** Detailed information of 10 RCT studies.

No.	Total sample	TG sample	TG effective	TG MoCA^a^	TG MMSE^b^	TG TCMSS^c^	CG sample	CG effective	CG MoCA^d^	CG MMSE^e^	CG TCMSS^f^
1	48	24	na	na	28.60±1.40	14.02±3.08	24	na	na	23.20±1.50	18.82±3.15
2	61	31	26	na	25.12±2.79	na	30	17	na	23.24±1.78	na
3	30	15	13	30.12±7.15	na	6.84±3.34	15	5	28.06±6.87	na	10.47±4.62
4	60	30	27	27.07±1.26	27.67±1.41	22.51±3.42	30	18	25.57±1.11	25.57±1.42	28.62±3.28
5	88	44	36	na	25.14±2.66	na	44	25	na	23.22±1.88	na
6	24	12	na	23.83±3.07	27.17±1.34	na	12	na	23.75±2.90	27.58±2.02	na
7	120	60	na	na	26.10±2.30	5.50±1.30	60	na	na	24.60±3.10	14.80±2.80
8	60	30	28	na	28.13±1.64	na	30	23	na	26.31±0.86	na
9	85	43	na	20.32±7.15	na	9.84±3.34	42	na	21.06±3.87	na	10.17±4.62

^a^,  ^b^,  ^c^,  ^d^,  ^e^,  ^f^: TG MoCA, TG MMSE, TG TCMSS, CG MoCA, CG MMSE and CG TCMSS are all continuous variable, and expressed in mean ± SD.

na, not available.

**Table 3:** Assessment of studies quality.

No.	Random allocation/random number table	Allocation concealment	Blinding description	Follow-up description	Score
1	YES/YES	NO	NO	NO	2
2	YES/NO	NO	UNCLEAR	YES	3
3	YES/YES	NO	UNCLEAR	NO	3
4	YES/NO	NO	NO	NO	1
5	YES/NO	NO	UNCLEAR	NO	2
6	YES/NO	NO	NO	YES	2
7	YES/NO	NO	NO	NO	1
8	YES/NO	NO	NO	NO	1
9	YES/ YES	NO	UNCLEAR	YES	4
